# Building resilience through self-defense: the role of martial arts in enhancing psychological strength among women

**DOI:** 10.3389/fpsyg.2025.1592326

**Published:** 2025-06-13

**Authors:** Aydın Pekel, Mehmet Behzat Turan, Meriç Eraslan, Mujahid Iqbal, Osman Pepe, Keziban Yoka, Osman Yoka

**Affiliations:** ^1^Department of Sports Management, Faculty of Sports Sciences, Marmara University, Istanbul, Türkiye; ^2^Faculty of Sports Sciences, Erciyes University, Kayseri, Türkiye; ^3^Faculty of Sports Sciences, Akdeniz University, Antalya, Türkiye; ^4^Beijing Key Laboratory of Applied Experimental Psychology, National Demonstration Center for Experimental Psychology Education (Beijing Normal University), Faculty of Psychology, Beijing Normal University, Beijing, China; ^5^Department of Sports Management, Faculty of Sports Sciences, Süleyman Demirel University, Isparta, Türkiye; ^6^Istanbul Esenyurt University, School of Physical Education and Sports, Niğde, Türkiye; ^7^Institute of Health Sciences, Erciyes University, Kayseri, Türkiye

**Keywords:** psychological resilience, women, violence, sport, martial arts

## Abstract

**Introduction:**

This study aimed to investigate differences in psychological resilience between women who participate in martial arts and those who do not, while also examining the influence of demographic and socioeconomic characteristics.

**Methods:**

A total of 802 women voluntarily participated, including 407 martial arts practitioners (Muay Thai, kickboxing, boxing, or taekwondo) and 395 women who practiced Pilates. Data were collected via an online self-report questionnaire that included the Psychological Resilience Scale and a demographic information form. Statistical analyses were conducted using descriptive statistics, independent sample t-tests, and one-way ANOVA.

**Results:**

Results showed that women engaged in martial arts demonstrated significantly higher levels of psychological resilience in the sub-dimensions of control (*p* < .01, *d* = 0.47) and challenge *p* < .01, *d* = 0.27) compared to non-practitioners. However, in the commitment sub-dimension, non-martial arts participants scored higher (*p* < .05, *d* = 0.35). Among martial artists, psychological resilience varied significantly based on age, experience of violence, and smoking status. Significant differences were found for non-martial artists according to age, educational attainment, and income level.

**Discussion:**

These findings suggest that participation in martial arts may enhance specific dimensions of psychological resilience, especially in managing stress and embracing challenges. However, it may not necessarily foster higher commitment levels.

## 1 Introduction

Combat sports have recently gained significant popularity among adults and children. This rise in interest can be attributed to the numerous benefits of martial arts, such as self-defense skills, enhanced mental well-being, and the harmony between mind and body (Zetaruk et al., 2000). Disciplines such as karate, kickboxing, boxing, Muay Thai, and taekwondo have garnered particular attention due to their structured techniques and guiding principles. Beyond their physical aspects, martial arts cultivate self-confidence, self-discipline, and the ability to focus and act decisively in various situations (Koçak and Balçıkanlı, 2021; Zengi, 2019). Researchers have highlighted the positive impact of martial arts on both physical and psychological health, noting that engaging in such activities fosters emotional regulation, socialization, and stress reduction by influencing neurophysiological processes, including the release of serotonin, dopamine, and noradrenaline during physical exertion (Duclos et al., 2003; Washington and Karen, 2001; Malmisur and Schempp, 2004; Rudd, 2005; Dumitriu, 2012; Kılıç, 2013; Kılıç and Aslan, 2016; Kılıç and Arslan, 2018). Given the well-established link between sports participation and psychological well-being (Akçakoyun et al., 2010; Tekin, 2008; Pasco et al., 2011), it is essential to explore the role of martial arts in fostering resilience and personal growth.

In Turkey, gender-based discourse and socialization are essential in shaping women’s opportunities, behaviors, and access to various areas, including sports. Gender perception is shaped according to the cultural and psychological characteristics of the society in which one lives (Bora, 2012; Koca, 2018). The literature has reported that sports can be considered one of the areas where gender norms are the strictest (Kavasoğlu and Yaşar, 2016). From the specific to the general, it is stated that the differences in sports-related experiences, successes, and performance characteristics of female and male athletes cannot be explained only by biological sex differences (Argut and Çelik, 2018; Koivula, 2001; Yuksel, 2014). The effect of gender perception on sports participation is included in the literature (Alley and Hicks, 2005; Yi-Hsiu and Chen-Yueh, 2013). Studies have reported that participation in sports is related to gender conformity based on social beliefs and perceptions (Chalabaev et al., 2013; Hardin and Greer, 2009). Accordingly, while men are more likely to participate in sports considered masculine than women, women are more likely to participate in sports considered feminine than men (Yi-Hsiu and Chen-Yueh, 2013). In Turkey, gender roles cause women to be directed to certain sports branches and to be excluded from others. For example, it has been concluded that football, boxing, taekwondo and wrestling are more suitable for men; gymnastics, aerobics, dance and yoga are ideal for women, and participation in these activities is shaped similarly (Kavasoğlu and Yaşar, 2016; Chalabaev et al., 2013). In Turkey, gender discrimination is expressed as women being deprived of basic needs, experiencing inequality in accessing opportunities and resources, being subjected to violence, and being represented at low rates in politics and business life. In other words, gender discrimination includes not being able to participate in decision-making mechanisms, being deprived of public opportunities, living in unhealthy conditions, being obstructed in working life, being harassed or mistreated at work, and being deprived of joining a union (Çolak, 2011). Various civil society organizations and projects operate in Turkey to eliminate these inequalities. For example, the Association for Sports and Physical Activity for Women (KASFAD), the United Nations Women’s Unit (UN Women) and Fenerbahçe Sports Club are collaborating and carrying out projects aiming to achieve gender equality in and through sports (UN Women Regional Office for Europe and Central Asia, 2022). In Turkey, gender affects women’s participation and representation in sports in various ways. Our work will support the prevention of violence against women who are involved in self-defense sports despite the gender perspective, increasing the number of women who are engaged in self-defense sports, women’s participation in sports, investment in women in sports, making people more sensitive to gender, including men in the process and developing gender equality in and through sports.

One of the most pressing societal concerns today is violence against women (VAW), a pervasive global public health issue that significantly affects women’s physical, emotional, and social well-being (Süner et al., 2024; Ersöz, 2016; Eygü and Nacaksız, 2024). Statistics indicate that approximately 736 million women worldwide experience intimate partner violence at some point in their lives (Süner et al., 2024). The consequences of such violence are profound, leading to mental health disorders such as depression, post-traumatic stress disorder, and anxiety, as well as physical issues like obesity, sleep disorders, substance abuse, and increased vulnerability to chronic illnesses (Yount et al., 2011; Al-Modallal, 2016; Roos et al., 2017; Bosch et al., 2017; Mason et al., 2012). VAW is also a critical concern in Turkey, where it encompasses various forms of physical, psychological, economic, and sexual violence stemming from deeply rooted gender inequalities. While legal frameworks such as Law No. 6284 on the Protection of Family and Prevention of Violence Against Women have been established to address this issue, societal attitudes and cultural norms continue to shape its prevalence and perception. Research suggests that traditional gender roles, patriarchal structures, and honor-based cultural expectations contribute to both the normalization and underreporting of violence against women (Ünver et al., 2024). Given these societal dynamics, exploring mechanisms that can empower women and enhance their psychological resilience is essential.

The current study draws on the Resilience Model of Richardson (2002), which conceptualizes resilience as a dynamic reintegration process following exposure to adversity. According to this model, resilience emerges when individuals face disruptions to their biopsychospiritual equilibrium (e.g., trauma, stress, life difficulties). They then engage internal and external protective factors to restore or enhance their functioning. Martial arts, which promote discipline, self-regulation, and personal mastery, may act as a resilience-building environment by helping practitioners respond adaptively to challenges. However, few studies have examined how specific subdimensions of resilience such as commitment, control, and challenge are influenced by martial arts training in women, particularly in the context of VAW or psychosocial stressors.

Extensive research on psychological resilience exists, covering areas such as psychotherapy, self-confidence, stress-coping strategies, sports commitment, athlete identity, and the effects of psychoeducation programs (Walsh, 2015; Şahin and Güçlü, 2018; Machisa et al., 2018; Döklü, 2018; Robbins et al., 2018; Mandıralı, 2019; Karademir and Açak, 2019; Xu et al., 2022; Kıcalı, 2024; Öner, 2024; Kutlu, 2024; Aslan, 2024). While resilience has been examined in terms of various factors, the role of sports (Hartmann et al., 2022), especially martial arts, has received limited attention in resilience research. Psychological resilience is a multidimensional and culturally influenced construct encompassing problem-solving abilities, self-regulation, determination, empathy, and an internal locus of control (Botou et al., 2017; Gürgan, 2006; Arslan and Ayas, 2019). Highly resilient individuals are adept at overcoming difficulties and demonstrate adaptability, strong self-efficacy, and openness to social support. Given these characteristics, martial arts may be a powerful tool for enhancing psychological resilience. In addition to martial arts participation, the study investigates the role of demographic and behavioral factors (e.g., age, income, education, and smoking) in shaping resilience. Prior studies have shown that smoking may co-occur with reduced coping ability or be used maladaptively to manage stress (Li et al., 2021). At the same time, higher income and educational attainment are often associated with stronger social support networks and psychological stability (Patel et al., 2018). This study aims to (1) compare the psychological resilience levels of women who practice martial arts with those who do not and (2) examine the impact of demographic factors (age, education level, income level, and smoking status) on resilience levels. The following research questions were examined within the scope of the study:

*RQ1*: Is there a significant difference in psychological resilience levels between female participants who practice martial arts and those who do not?

*RQ1a*: Does the difference in psychological resilience between martial arts practitioners and non-practitioners vary by education level?

*RQ1b*: Does the difference in psychological resilience between martial arts practitioners and non-practitioners vary by income level?

*RQ1c*: Does the difference in psychological resilience between martial arts practitioners and non-practitioners vary by smoking status?

*RQ1d*: Does the difference in psychological resilience between martial arts practitioners and non-practitioners vary by age?

*RQ1e*: Does the difference in psychological resilience between martial arts practitioners and non-practitioners vary by the type of violence experienced?

**Figure 1:**
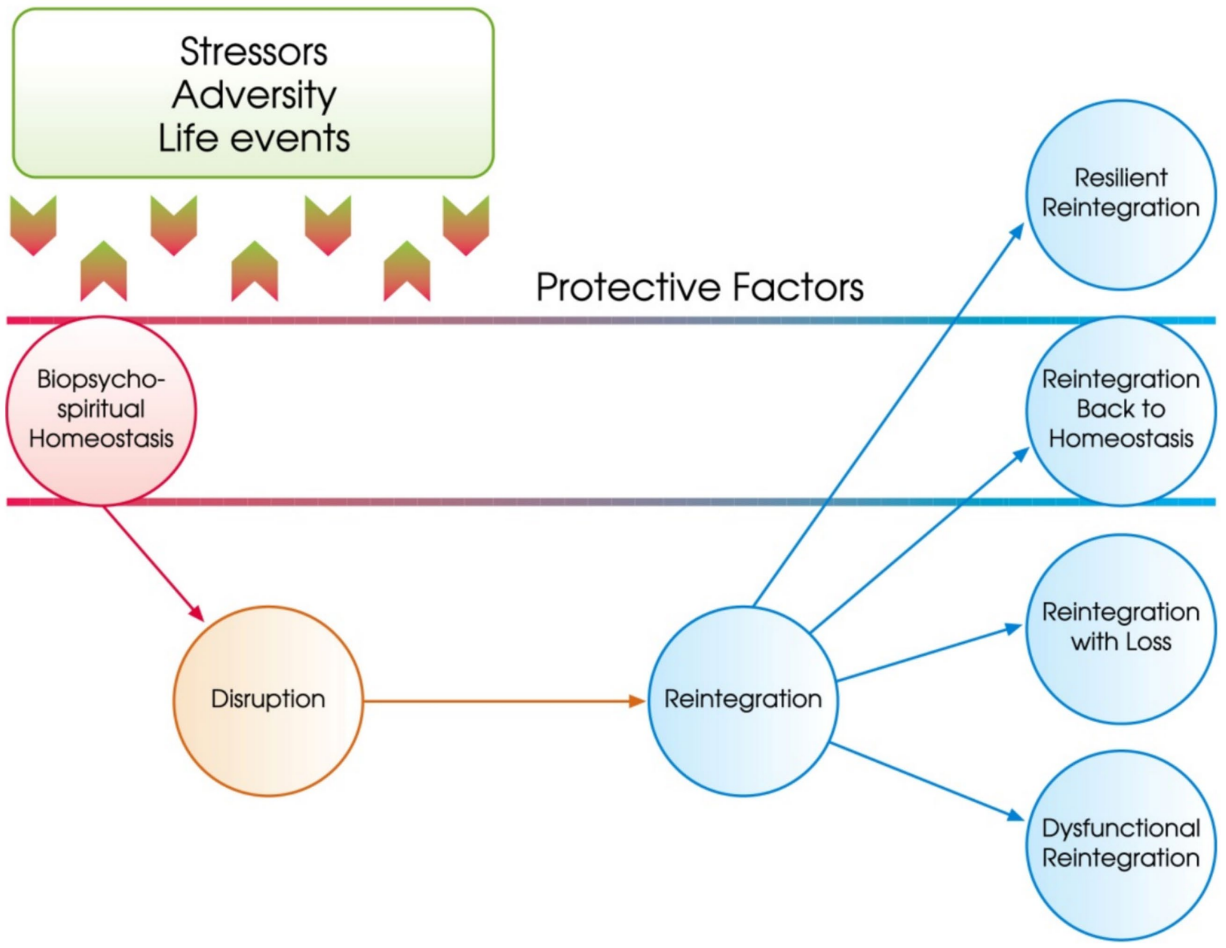
The resilience model.

The resilience model is presented in Figure 1.

## 2 Materials and methods

### 2.1 Data collection

The Ethics Committee of Erciyes University, Social and Human Sciences Unit approved the study on 24/09/2024 (Application No. 427). The research population consists of women attending martial arts training courses in Turkey. The sample comprised female participants over the age of 18, selected through convenience sampling. This non-probability method allowed the researchers to recruit participants who were readily accessible and met the basic inclusion criteria relevant to the study’s objectives (Büyüköztürk et al., 2012; Karasar, 2016). Gym owners and martial arts training centers were contacted to recruit participants, and approximately 110 centers agreed to facilitate the study. With the approval of gym managers, the study’s purpose and objectives were explained to potential participants. A total of 407 women who met the eligibility criteria voluntarily participated. Additionally, a comparison group of 395 women who practiced Pilates for at least 3 years and had no health issues was included in the study.

**Figure 2:**
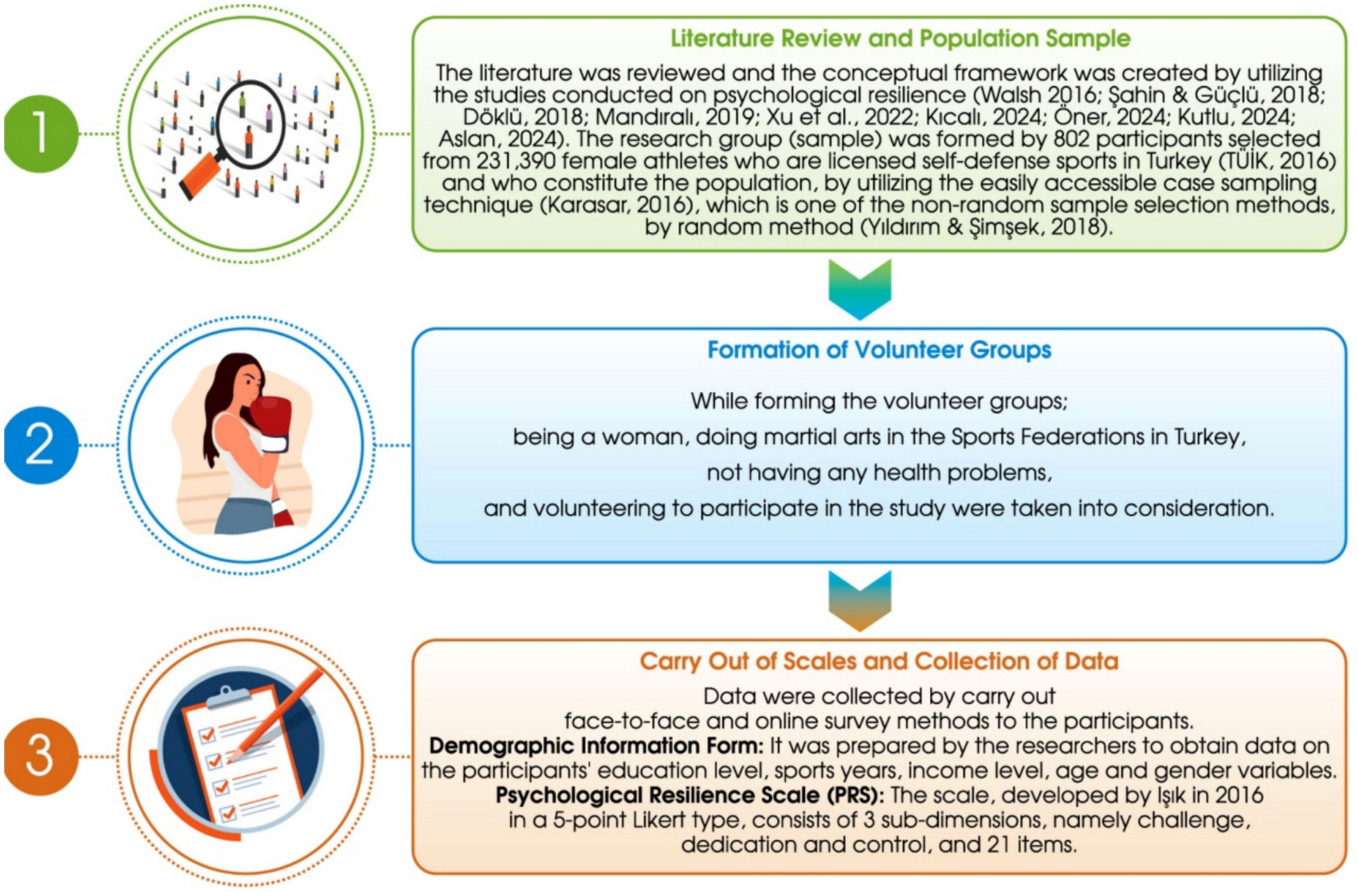
Flow chart of sample selection and procedure.

Data was collected online using social web-based applications such as WhatsApp and email. Surveys were administered through Google Forms,^1^ ensuring accessibility and ease of completion. Participants were informed about the study’s nature, objectives, and confidentiality. All responses were anonymous, and no personally identifiable information (e.g., names, IP addresses, or contact details) was collected. Google Forms was set to not collect email addresses, ensuring that data remained anonymous. They were assured that their data would be used solely for research purposes and that they could withdraw their participation at any stage, even after submitting the survey. Informed consent was obtained electronically before participation. The researcher provided clear instructions on completing the study, explained the rating scale, and addressed any participant queries. Although the survey was not time-restricted, participants typically took 8–10 min to complete. Responses were reviewed for completeness upon submission, and any inconsistencies, such as double-marked answers, were identified and addressed. Participants were not offered any compensation for their participation but were thanked for their time and voluntary contribution to the study. Data collection took place from September 2024 to January 2025. For the data collection procedure, see Figure 2.

**Figure 3:**
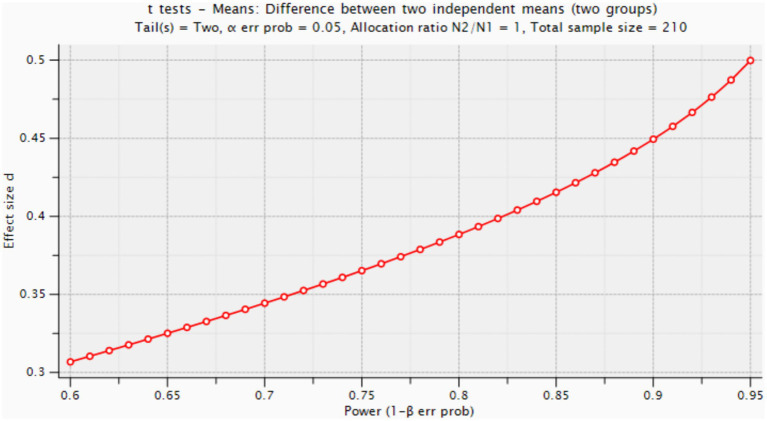
G*Power analyses.

To ensure an adequate sample size, a G*Power analysis was conducted. It determined that a minimum of 420 participants (210 martial arts practitioners and 210 Pilates practitioners) were required for the study, with a statistical power of 0.80, an effect size of 0.5, and an error margin of 0.05 (Figure 3). Given the final sample size, the study meets the necessary statistical requirements (Sezgin, 2012).

### 2.2 Inclusion criteria

Participants in this study must meet the following criteria: they must be female and aged 18 years or older. They must be actively involved in at least one of the following martial arts disciplines: Muay Thai, kickboxing, boxing, or taekwondo. Additionally, participants were required to have at least 3 years of amateur martial arts experience. This duration was chosen based on previous literature suggesting that long-term engagement in physical activity is positively associated with psychological resilience and emotional well-being (Kim et al., 2023; Arida and Teixeira-Machado, 2021). Participants must not have any current health conditions or physical impairments that would prevent their participation in martial arts training or the research study. Furthermore, participants must have experienced at least one form of violence—whether physical, verbal, economic, emotional, or sexual—during their lifetime, which is essential for addressing the relationship between martial arts training and resilience in the context of violence.

### 2.3 Measures

#### 2.3.1 Demographic information form

The researcher developed a structured demographic information form to collect key participant details, including age, education level, income level, and other relevant socio-demographic variables.

#### 2.3.2 Exposure to violence

In this study, exposure to violence was self-reported by the participants through a survey that included questions regarding their experiences with different forms of violence (e.g., physical, emotional, economic). Participants participated in the study by stating that they had been subjected to violence, according to their statements.

#### 2.3.3 Psychological Resilience Scale

The Psychological Resilience Scale developed by Işık (2016) is a 21-item, 5-point Likert-type measurement tool designed to assess individuals’ psychological hardiness. The scale is grounded in Kobasa’s theoretical framework of hardiness and comprises three core sub-dimensions: challenge, commitment, and control. Each item is rated on a scale from 1 (strongly disagree) to 5 (strongly agree), with higher scores indicating greater psychological resilience (Işık, 2016).

The challenge sub-dimension (items 7, 8, 9, 13, 14, 16, and 17) evaluates an individual’s tendency to view stressors and adversities as opportunities for personal growth and development. Individuals scoring high in this domain are more likely to perceive difficult circumstances not as threats, but as stimulating challenges that foster learning and adaptation.

The commitment sub-dimension (items 1, 2, 3, 5, 6, 18, and 21) measures the extent to which individuals are engaged with and committed to their goals, values, and responsibilities, even under challenging conditions. This component reflects a sense of purpose and persistence in the face of adversity.

The control sub-dimension (items 4, 10, 11, 12, 15, 19, and 20) assesses individuals’ perceived ability to influence and regulate events in their lives. It reflects a sense of internal locus of control and confidence in one’s problem-solving capacities. Items 2 and 15 are reverse-scored to minimize response bias and promote more accurate evaluation of psychological constructs.

The Cronbach alpha coefficient for the whole scale was found to be. 76, whereas the values of Cronbach alpha coefficient for dimension factors of the scale ranged between 0.62 and 0.74. The findings of the study revealed that the scale was a valid and reliable instrument for measuring psychological hardiness personality trait.

To establish the construct validity of the scale, both exploratory factor analysis (EFA) and confirmatory factor analysis (CFA) were performed. EFA results revealed a three-factor structure consistent with theoretical expectations, with all 21 items loading significantly onto one of the three dimensions. Based on factor loadings and conceptual alignment, these factors were named commitment, control, and challenge. The cumulative variance explained by the three factors indicated that the scale adequately captured the multidimensional nature of psychological resilience.

Following EFA, CFA was conducted to test the fit of the three-factor model. The CFA results confirmed the model’s suitability, with acceptable goodness-of-fit indices supporting the scale’s factorial structure. Additionally, item discrimination analyses were conducted using independent samples t-tests comparing the upper 27% and lower 27% scoring groups. Statistically significant differences were observed for all items (*p* < 0.05), indicating strong item-level discrimination and supporting the scale’s ability to differentiate between individuals with high and low levels of psychological resilience.

Overall, the findings of the study provide robust evidence for the scale’s reliability and validity as an instrument for assessing psychological resilience traits in individuals. The scale offers a theoretically grounded, psychometrically sound tool suitable for use in both research and applied settings, including educational, clinical, and organizational contexts.

The goodness-of-fit indices of the Psychological Resilience Scale are presented in Table 1.

**Table 1:** Goodness-of-fit indices of the psychological resilience scale.

χ2	df	χ2/df	RMR	CFI	GFI	RMSEA
226.32	82	2.76	0.06	0.91	0.89	0.06

In the present study, the scale demonstrated adequate internal consistency. The overall Cronbach’s alpha coefficient for the entire scale was calculated as 0.76, indicating a reliable level of internal consistency. Sub-dimension reliability coefficients were found to be 0.81 for challenge, 0.75 for commitment, and 0.89 for control, all of which suggest satisfactory to high internal reliability.

### 2.4 Statistical analysis

We first examined the data distribution using the Kolmogorov–Smirnov test and reviewed the skewness and kurtosis values to determine the appropriate statistical tests for the data analysis. According to George and Mallery (2003), values within the ±2 range are acceptable for meeting the normality assumption. Our skewness and kurtosis values fell within these acceptable limits (see Table 2), indicating that the data approximately follows a normal distribution. Before proceeding with the analyses, we conducted a G*Power analysis to determine the required sample size to achieve sufficient statistical power for detecting meaningful differences between groups. The power analysis indicated that our sample size was adequate to detect medium-sized effects at a significance level of *p* < 0.05 with a power of 0.80.

**Table 2:** Descriptive statistics and frequency values.

		Martial arts athlete		Not a martial arts athlete	
Variable	Category	*n*	*%*	*n*	*%*
Income level	20.000 and below	146	35.9	149	37.7
	20,001–30,000	60	14.7	48	12.2
	30,001–40,000	87	21.4	85	21.5
	40,001–50,000	61	15	59	14.9
	50,001 and above	53	13	54	13.7
Age	18–21	124	30.5	126	31.9
	22–24	153	37.6	140	35.4
	25–27	74	18.2	63	15.9
	28–31	56	13.8	66	16.7
Education level	High school	102	25.1	75	19
	University	248	60.9	263	66.6
	College	57	14	57	14.4
Smoking status	Yes	121	29,7	121	30.6
	No	286	70,3	274	69.4
Type of violence	Physically	129	31.7	99	25.1
	Oral	158	38.8	147	37.2
	Emotional	48	11.8	76	19.2
	Economic	48	11.8	42	10.6
	Sexual	24	5.9	31	7.8
Branch	Kickboxing	99	24.3	Pilates	
	Boxing	104	25.6		
	Muay Thai	96	23.6		
	Taekwondo	108	26.5		
Total	407			395	

We calculated Cronbach’s alpha for the psychological resilience scale and its subscales for reliability. Cronbach’s alpha for the overall scale was 0.84, indicating good internal consistency. To assess differences between groups, we applied *t*-tests for two-group comparisons and one-way ANOVA for comparing more than two groups. When significant results were found in the ANOVA, we conducted post-hoc pairwise comparisons using the LSD (Least Significant Difference) test to identify which specific group differences contributed to the overall significant effect. Additionally, we reported effect sizes (Cohen’s *d* for t-tests and partial eta squared for ANOVA) to provide an understanding of the magnitude of the differences between groups. Effect sizes were interpreted based on Cohen’s conventions: small (*d* = 0.20 or partial eta squared = 0.01), medium (*d* = 0.50 or partial eta squared = 0.06), and large (*d* = 0.80 or partial eta squared = 0.14). All analyses were performed using IBM SPSS Statistics 27, and the significance level for all tests was set at *p* < 0.05.

## 3 Results

### 3.1 Descriptive statistics and frequency values

Table 2 presents the descriptive statistics and frequency distribution for various demographic variables. Regarding income level, 35.9% of martial arts practitioners earn 20,000 TL or below, compared to 37.7% of non-practitioners. A similar trend is observed in higher income brackets, with 13% of martial arts participants and 13.7% of non-practitioners earning 50,000 TL or more. The age distribution shows that the largest group of martial arts practitioners (37.6%) is between 22 and 24 years old, followed by 30.5% aged 18 to 21. Non-practitioners show a similar trend, with 31.9% in the 18–21 age range and 35.4% between 22 and 24. Regarding education level, 60.9% of martial arts participants hold a university degree, while 66.6% of non-practitioners have the same level of education. Interestingly, 25.1% of martial arts participants have a high school education compared to 19% of non-practitioners. Concerning smoking status, 29.7% of martial arts participants smoke, which is nearly identical to 30.6% of non-practitioners who reported smoking. In terms of exposure to violence, martial arts practitioners report higher levels of physical violence (31.7%) compared to non-practitioners (25.1%). Verbal violence is reported by 38.8% of martial arts participants and 37.2% of non-practitioners. Emotional violence is reported by 11.8% of martial arts participants and 19.2% of non-practitioners. Finally, martial arts practitioners report engagement in various disciplines, with 24.3% participating in kickboxing, 25.6% in boxing, 23.6% in Muay Thai, and 26.5% in taekwondo.

### 3.2 Normality distribution and reliability

Table 3 presents the normality distributions and reliability for the psychological resilience scores of female participants who do and do not practice martial arts. The data are reported across three sub-dimensions of psychological resilience: Commitment, Control, and Challenge. For participants who engage in martial arts, the mean score for Commitment was 18.36 (*SD* = 2.31), skewness of 0.017 and kurtosis of 0.474, indicating that the distribution is nearly symmetrical and slightly platykurtic. The Control sub-dimension had a mean score of 21.96 (*SD* = 2.54), skewness of 0.265, and kurtosis of 0.479, suggesting a slight positive skew but still close to a normal distribution. The Challenge sub-dimension showed a mean score of 21.83 (*SD* = 3.22), skewness of −0.258, and kurtosis of 0.158, indicating a slightly negative skew and a near-normal distribution. For participants who do not engage in martial arts, the Commitment sub-dimension had a mean score of 19.26 (*SD* = 2.81), with skewness of 0.297 and kurtosis of 0.253, suggesting a moderately positive skew and a nearly normal distribution. The mean score for the Control sub-dimension was 20.82 (*SD* = 2.30), with skewness of 0.048 and kurtosis of −0.181, indicating a near-normal distribution with a slight negative kurtosis. Finally, the Challenge sub-dimension had a mean score of 20.90 (*SD* = 3.56), skewness of 0.155, and kurtosis of −0.123, showing a slightly positive skew and a near-normal distribution.

**Table 3:** Normality distributions and reliability scores of psychological resilience for female participants who do and do not do martial arts.

Martial arts status	Scale sub-dimension	*N*	Min	Max	M	SD	Skewness	Kurtosis	Cronbach’s alpha
Yes	Commitment	407	12.00	26.00	18.36	2.310	0.017	0.474	0.752
	Control	407	16.00	29.00	21.96	2.535	0.265	0.479	0.892
	Challenge	407	13.00	28.00	21.830	3.216	−0.258	0.158	0.812
No	Commitment	395	12.00	27.00	19.26	2.814	0.297	0.253	0.710
	Control	395	16.00	26.00	20.82	2.295	0.048	−0.181	0.845
	Challenge	395	13.00	28.00	20.90	3.564	0.155	−0.123	0.774

Cronbach’s alpha values for each sub-dimension indicate good internal consistency across the resilience measures, with the Commitment, Control, and Challenge sub-dimensions for both groups having alpha values ranging from 0.710 to 0.892. These values suggest that the scales have acceptable to excellent reliability across both groups of participants.

### 3.3 Differences in psychological resilience scores between martial arts practitioners and non-practitioners

The comparison of psychological resilience scores between female participants who do and do not practice martial arts revealed statistically significant differences across all three sub-dimensions: commitment, control, and challenge (*p* < 0.01 for all). Surprisingly, non-martial arts participants scored higher on the *commitment* sub-dimension (*M* = 19.26, *SD* = 2.81) compared to those who practice martial arts (*M* = 18.36, *SD* = 2.31), with a small effect size (Cohen’s *d* = 0.35). This finding contrasts with the common assumption that martial arts training enhances psychological resilience across all dimensions. It may suggest that individuals not involved in martial arts may rely more on intrinsic motivation or personal routines unrelated to physical discipline. However, further qualitative or longitudinal research is needed to explore this interpretation.

In contrast, participants involved in martial arts showed significantly higher scores on the control (*M* = 21.96) and challenge (*M* = 21.83) dimensions than non-practitioners. The effect size was moderate for control (Cohen’s *d* = 0.47) and trim for the challenge (Cohen’s *d* = 0.27), indicating that martial arts training may contribute more strongly to enhancing one’s ability to regulate behavior and perceive stressful situations as growth opportunities. These results suggest that participation in martial arts is associated with improved resilience in managing challenges and maintaining control. However, the unexpected finding on commitment underscores the need for deeper investigation into how various forms of activity, including non-physical domains, contribute to different aspects of resilience (Table 4).

**Table 4:** Comparison of psychological resilience scores based on martial arts participation.

Scale sub-dimension	Martial arts status	*N*	M	SD	t	F	*p*	Cohen’s d
Commitment	Yes	407	18.36	2.31	−4.99	14.74	**0.002**	0.35
	No	395	19.26	2.81				
Control	Yes	407	21.96	2.53	6.67	1.83	**0.005**	0.47
	No	395	20.82	2.29				
Challenge	Yes	407	21.83	3.21	3.84	2.80	**0.007**	0.27
	No	395	20.90	3.56				

^*^p < 0.05, ^**^p < 0.01; Cohen’s d = 0.20 = Small effect, 0.50 = Medium effect, 0.80 or higher = Large effect.

### 3.4 Comparison of psychological resilience scores of participants according to age

A comparison of psychological resilience scores by age group for martial arts practitioners and non-practitioners across the Commitment, Control, and Challenge sub-dimensions was conducted. For Commitment, no significant age differences were observed for martial arts practitioners [*F*(3, 407) = 0.440, *p* = 0.724]. However, for non-practitioners, significant differences were found [*F*(3, 395) = 2.704, *p* = 0.04, η^2^ = 0.020], with the 25–27 age group scoring higher than the 18–21 age group (*p* = 0.020). In Control, martial arts practitioners showed significant differences [*F*(3, 407) = 0.4309, *p* = 0.00, η^2^ = 0.031], with the 22–24 age group scoring higher than the 25–27 (*p* = 0.031) and 28–31 age groups (*p* = 0.022). Non-practitioners also exhibited significant differences [*F*(3, 395) = 2.996, *p* = 0.03, η^2^ = 0.022], with the 22–24 age group scoring higher than the 18–21 age group (*p* = 0.022). For Challenge, significant differences were found for both groups. Martial arts practitioners showed differences [*F*(3, 407) = 3.500, *p* = 0.01, η^2^ = 0.025], with the 18–21 age group scoring higher than the 22–24 age group (*p* = 0.025). Non-practitioners also displayed significant differences [*F*(3, 395) = 3.500, *p* = 0.01, η^2^ = 0.026], with the 18–21 age group scoring higher than the 22–24 age group (*p* = 0.026). These findings indicate that age affects resilience scores, especially for non-practitioners, with martial arts practitioners showing more consistent resilience scores across age groups (Table 5).

**Table 5:** Comparison of psychological resilience scores of participants according to age.

Martial arts status	Scale sub-dimension	Age	*N*	M	SD	F	*p*	Lsd	η^2^
Yes	Commitment	18–21^a^	124	18.39	1.74	0.440	0.724		
		22–24^b^	153	18.20	2.68				
		25–27^c^	74	18.51	2.31				
		28–31^d^	56	18.51	2.33				
	Control	18–21^a^	124	21.61	2.21	0.4309	**0.003**	b*a	
		22–24^b^	153	22.54	2.83			b*c	0.031
		25–27^c^	74	21.59	2.23			b*d	
		28–31^d^	56	21.67	2.49			d*a	
	Challenge	18–21^a^	124	22.59	3.30	3.500	**0.001**	a*b	0.025
		22–24^b^	153	21.44	3.14			a*c	
		25–27^c^	74	21.63	3.30			a*d	
		28–31^d^	56	21.44	2.86			c*b	
No	Commitment	18–21^a^	126	19.40	2.75	2.704	**0.04**	c*a	0.020
		22–24^b^	140	19.39	2.83			c*b	
		25–27^c^	63	19.63	2.63			c*d	
		28–31^d^	66	18.39	2.95			b*d	
	Control	18–21^a^	126	20.71	2.11	2.996	**0.03**	b*a	
		22–24^b^	140	21.26	2.41			b*c	0.022
		25–27^c^	63	20.57	2.19			c*d	
		28–31^d^	66	20.36	2.34			a*c	
	Challenge	18–21^a^	126	20.86	3.69	3.500	**0.01**	b*a	0.026
		22–24^b^	140	21.47	3.46			b*c	
		25–27^c^	63	20.92	3.82			b*d	
		28–31^d^	66	19.77	3.01				

^*^p < 0.05, ^**^p < 0.01, η2 = 0.01 = Small effect 0.06 = Medium effect 0.14 = Large effect. Bold values indicate statistically significant results (*p* < 0.05). Different lowercase letters (a, b, c, d) next to group means indicate statistically significant differences between those groups based on post hoc LSD tests (*p* < 0.05). Groups sharing the same letter are not significantly different.

### 3.5 Comparison of psychological resilience scores of participants according to the education level

No significant differences were found in Commitment scores across education levels for martial arts practitioners [*F*(2, 407) = 2.724, *p* = 0.06]. However, for non-practitioners, significant differences were observed in Commitment [*F*(2, 395) = 3.964, *p* = 0.02, η^2^ = 0.020]. Post-hoc comparisons revealed that participants with a college education scored significantly lower than those with undergraduate education (*p* = 0.02) and high school education (*p* = 0.020). In Control, for non-practitioners, significant differences were found [*F*(2, 395) = 3.670, *p* = 0.02, η^2^ = 0.018]. Post-hoc tests revealed that participants with a college education scored significantly higher than those with a high school education (*p* = 0.018). For Challenge, non-practitioners showed significant differences [*F*(2, 395) = 5.943, *p* = 0.00, η^2^ = 0.029]. Post-hoc analyses showed that participants with high school education scored significantly lower than those with undergraduate education (*p* = 0.029) and college education (*p* = 0.029) (Table 6).

**Table 6:** Comparison of psychological resilience scores of participants according to the education level variable.

Martial arts status	Scale sub-dimension	Education level	*N*	M	SD	F	*p*	LSD	η^2^
Yes	Commitment	High school^a^	102	17.95	2.30	2.72	0.06		
		College^b^	57	18.78	1.97				
		Undergraduate^c^	248	18.43	2.36				
	Control	High school^a^	102	22.02	3.01	0.36	0.69		
		College^b^	57	21.70	2.03				
		Undergraduate^c^	248	22.00	2.42				
	Challenge	High school^a^	102	21.47	3.29	0.91	0.40		
		College^b^	57	22.08	2.97				
		Undergraduate^c^	248	21.91	3.23				
No	Commitment	High school^a^	75	19.30	3.52	3.96	**0.02**	c*a	0.020
		College^b^	57	18.31	3.08			c*b	
		Undergraduate^c^	263	19.46	2.47			a*b	
	Control	High school^a^	75	20.18	2.27	3.67	**0.02**	b*a	0.018
		College^b^	57	21.01	2.64			b*c	
		Undergraduate^c^	263	20.96	2.19			c*a	
	Challenge	High school^a^	75	19.66	4.14	5.94	**0.008**	c*a	0.029
		College^b^	57	20.94	4.13			c*b	
		Undergraduate^c^	263	21.25	3.16			b*a	

^*^p < 0.05, ^**^p < 0.01, η^2^ = 0.01 = Small effect, 0.06 = Medium effect, 0.14 = Large effect. Bold values indicate statistically significant results (*p* < 0.05). Different lowercase letters (a, b, c, d) next to group means indicate statistically significant differences between those groups based on post hoc LSD tests (*p* < 0.05). Groups sharing the same letter are not significantly different.

### 3.6 Comparison of psychological resilience scores of participants according to the income level

For non-practitioners, significant differences were observed in Commitment [*F*(4, 395) = 9.592, *p* = 0.00, η^2^ = 0.090]. Participants with higher income levels scored significantly higher than those with lower ones. In Control, significant differences were found for non-practitioners [*F*(4, 395) = 3.134, *p* = 0.01, η^2^ = 0.031]. Participants with higher income levels scored significantly higher than those with lower ones. For Challenge, non-practitioners showed significant differences [*F*(4, 395) = 3.428, *p* = 0.00, η^2^ = 0.034]. Participants with lower income levels scored significantly higher than those with higher ones. For martial arts practitioners, significant differences were found in Challenge [*F*(4, 407) = 2.325, *p* = 0.05, η^2^ = 0.023]. Participants with lower income levels scored significantly higher than those with higher (Table 7).

**Table 7:** Comparison of psychological resilience scores of participants according to the income level.

Martial arts status	Psychological resilience	Income Level	*N*	M	SD	F	*p*	LSD	η^2^
Yes	Commitment	20,000 and below^a^	146	18.49	2.28	0.61	0.65		
		20,001–30,000^b^	60	18.38	1.87				
		30,001–40,000^c^	87	18.21	2.24				
		40,001–50,000^d^	61	18.54	2.39				
		50,001 and above^e^	53	18.00	2.81				
	Control	20,000 and below^a^	146	21.97	2.70	1.11	0.34		
		20,001–30,000^b^	60	22.56	3.03				
		30,001–40,000^c^	87	21.82	2.40				
		40,001–50,000^d^	61	21.80	2.01				
		50,001 and above^e^	53	21.69	2.12				
	Challenge	20,000 and below^a^	146	22.43	3.04	2.32	**0.05**	a*b	
		20,001–30,000^b^	60	21.58	3.16			a*c	0.023
		30,001–40,000^c^	87	21.37	2.89			a*d	
		40,001–50,000^d^	61	21.26	3.53			a*e	
		50,001 and above^e^	53	21.83	3.66			b*c	
No	Commitment	20,000 and below^a^	149	19.71	2.75	9.59	**0.004**	e*a	0.090
		20,001–30,000^b^	48	18.04	2.18			a*b	
		30,001–40,000^c^	85	18.25	2.57			e*c	
		40,001–50,000^d^	59	19.40	2.68			e*d	
		50,001 and above^e^	54	20.55	3.12			a*d	
	Control	20,000 and below^a^	149	20.70	1.98	3.13	**0.01**	d*a	0.031
		20,001–30,000^b^	48	20.93	2.52			d*b	
		30,001–40,000^c^	85	20.43	2.56			d*c	
		40,001–50,000^d^	59	21.72	2.39			d*e	
		50,001 ve üzeri^e^	54	20.70	2.12			a*c	
	Challenge	20,000 and below^a^	149	21.71	3.35	3.42	**0.007**	a*b	0.034
		20,001–30,000^b^	48	20.77	3.15			a*c	
		30,001–40,000^c^	85	20.12	4.02			a*d	
		40,001–50,000^d^	59	20.62	3.94			a*e	
		50,001 and above^e^	54	20.35	2.8				

^*^p < 0.05, ^**^p < 0.01, η^2^ = 0.01 = Small effect, 0.06 = Medium effect, 0.14 = Large effect. Bold values indicate statistically significant results (*p* < 0.05). Different lowercase letters (a, b, c, d) next to group means indicate statistically significant differences between those groups based on post hoc LSD tests (*p* < 0.05). Groups sharing the same letter are not significantly different.

### 3.7 Comparison of psychological resilience scores of participants according to smoking status

Smoking behavior was examined in this study as it is often considered a maladaptive coping mechanism, which may be inversely related to psychological resilience. For martial arts practitioners, significant differences were observed in the Control sub-dimension of resilience [*F*(1, 406) = 3.375, *p* = 0.02, Cohen’s *d* = 0.23], with smokers scoring significantly lower than non-smokers. This suggests that martial artists who avoid smoking may exhibit stronger control over their responses to stress and adversity. However, for non-practitioners, no significant differences were found in the Commitment, Control, or Challenge sub-dimensions based on smoking status (Table 8). These results support the notion that long-term engagement in martial arts, combined with healthier lifestyle choices, may contribute to enhanced psychological resilience.

**Table 8:** Comparison of psychological resilience scores of participants according to smoking status.

Martial arts status	Scale	Smoking status	*N*	M	SD	F	*p*	t	Cohen’s d
Yes	Commitment	Yes	121	18.50	2.20	0.441	0.41	0.881	
		No	286	18.30	2.35				
	Control	Yes	121	21.54	2.09	3.375	**0.02**	–2.197	0.23
		No	286	22.14	2.68				
	Challenge	Yes	121	21.84	3.18	0.000	0.95	0.051	
		No	286	21.82	3.23				
No	Commitment	Yes	121	19.55	3.18	13.386	0.18	1.34	
		No	274	19.14	2.62				
	Control	Yes	121	20.88	2.12	0.797	0.74	0.324	
			274	20.80	2.37				
	Challenge	Yes	121	20.61	2.98	11.830	0.27	−1.102	
		No	274	21.04	3.79				

^*^p < 0.05, ^**^p < 0.01, Cohen’s d = 0.20 = Small effect, 0.50 = Medium effect, 0.80 or higher = Large effect. Bold values indicate statistically significant results (*p* < 0.05).

### 3.8 Comparison of participants’ psychological resilience scores according to the type of violence

For non-practitioners, significant differences were observed in the Commitment scores [*F*(4, 397) = 25.430, *p* = 0.00, η^2^ = 0.207], with participants exposed to sexual violence scoring significantly higher than those exposed to physical, moral, emotional, and economic violence. Similarly, for Control, significant differences were found [*F*(4, 397) = 3.311, *p* = 0.01, η^2^ = 0.033], with participants exposed to physical violence scoring significantly lower than those exposed to emotional violence. For Challenge, significant differences were observed [*F*(4, 397) = 5.701, *p* = 0.00, η^2^ = 0.055], with participants exposed to sexual violence scoring significantly higher than those exposed to physical, moral, emotional, and economic violence. For martial arts practitioners, significant differences were observed in the Commitment scores [*F*(4, 395) = 2.995, *p* = 0.02, η^2^ = 0.209], with participants exposed to sexual violence scoring significantly higher than those exposed to physical, moral, emotional, and economic violence. However, no significant differences were found in the Control or Challenge sub-dimensions for martial arts practitioners (Table 9).

**Table 9:** Comparison of participants’ psychological resilience scores according to the type of violence.

Martial arts status	Scale sub-dimension	Type of violence	*N*	M	SD	F	*p*	LSD	η^2^
Yes	Commitment	Physically^a^	129	18.12	1.99	2.995	**0.02**	e*a	
		Oral^b^	158	18.20	2.28			e*b	0.209
		Emotional^c^	48	18.35	2.72			e*c	
		Economic^d^	48	18.87	2.24			e*d	
		Sexual^e^	24	19.62	2.85				
	Control	Physically^a^	129	22.34	3.08	1.980	0.09		
		Oral^b^	158	21.70	2.42				
		Emotional^c^	48	22.31	2.04				
		Economic^d^	48	21.89	1.97				
		Sexual^e^	24	21.16	1.20				
	Challenge	Physically^a^	129	21.42	3.43	1.784	0.13		
		Oral^b^	158	22.27	3.42				
		Emotional^c^	48	21.20	2.71				
		Economic^d^	48	22.02	2.91				
		Sexual^e^	24	21.91	1.17				
No	Commitment	Physically^a^	99	18.39	1.76	25.430	**0.002**	e*a	
		Oral^b^	147	18.65	2.28			e*b	0.207
		Emotional^c^	76	19.55	3.20			e*c	
		Economic^d^	42	20.02	3.05			e*d	
		Sexual^e^	31	23.22	2.92				
	Control	Physically^a^	99	20.78	2.08	3.311	**0.01**	c*a	
		Oral^b^	147	21.04	2.24			a*b	
		Emotional^c^	76	21.22	2.46			c*d	0.033
		Economic^d^	42	19.83	2.54			c*e	
		Sexual^e^	31	20.32	2.03				
	Challenge	Physically^a^	99	21.36	3.31	5.701	**0.005**	e*a	0.055
		Oral^b^	147	20.40	3.61			e*b	
		Emotional^c^	76	21.88	3.62			e*c	
		Economic^d^	42	19.19	3.11			e*d	
		Sexual^e^	31	21.77	3.44				

^*^p < 0.05, ^**^p < 0.01, η^2^ = 0.01 = Small effect, 0.06 = Medium effect, 0.14 = Large effect. Bold values indicate statistically significant results (*p* < 0.05). Different lowercase letters (a, b, c, d) next to group means indicate statistically significant differences between those groups based on post hoc LSD tests (*p* < 0.05). Groups sharing the same letter are not significantly different.

## 4 Discussion

Today, all types of violence negatively impact human life, with violence against women standing out as one of the most pressing societal concerns. Beyond the physical harm it causes, the psychological consequences of violence, such as trauma, anxiety, and diminished self-esteem, make it a crucial subject for research. At the same time, violence is a phenomenon that undermines psychological resilience, making it essential to examine factors that may strengthen resilience in affected individuals (Al-Modallal, 2016). In line with this perspective, this study explores whether participation in combat sports influences the psychological resilience of female martial artists compared to non-practitioners.

Examining the psychological resilience scores of female participants who engaged in martial arts versus those who did not reveal significant differences across several sub-dimensions, namely commitment, control, and challenge. Specifically, individuals who did not practice martial arts exhibited higher levels of commitment, whereas those who participated in martial arts scored higher in the sub-dimensions of control and challenge. While participants in martial arts exhibited significantly higher levels of psychological resilience in the sub-dimensions of control and challenge, the control group showed more significant commitment. This finding contradicts the hypothesis that martial arts would enhance resilience uniformly across all sub-dimensions. One possible interpretation of this contradiction is that the intense nature of martial arts training could lead to burnout, potentially reducing commitment levels. Participants in martial arts may experience physical and mental fatigue due to the rigorous demands of the sport, which could adversely affect their sustained engagement and influence their resilience in this area. Additionally, given that participants in this study have experienced various forms of trauma, certain aspects of martial arts training such as physical contact, sparring, or exposure to aggressive environments may act as psychological triggers. These elements could contribute to discomfort or avoidance responses, thereby diminishing commitment despite the resilience-building potential of martial arts.

On the other hand, the higher commitment levels observed in the control group, which primarily consisted of Pilates practitioners, may reflect the unique characteristics of this practice. Pilates, a low-impact, mindfulness-oriented exercise, might foster a more sustainable and less stressful commitment to physical activity, enhancing participants’ resilience in this dimension. This suggests that the type of physical activity and the associated social and cultural contexts may be crucial in shaping individuals’ psychological resilience. Given that Pilates emphasizes balance, mindfulness, and body awareness, participants might be more likely to experience positive psychological outcomes, contributing to their higher levels of commitment. Previous research suggests that participation in sports fosters improved coping mechanisms and reduces pessimistic psychological patterns associated with monotonous or purposeless living conditions (Küçük and Koç, 2004). Şahin et al. (2012) highlight that physical activities, including sports, enhance individuals’ ability to navigate life’s challenges, thereby increasing physical resilience. Further studies, such as Karademir and Açak (2019), have shown that individuals participating in individual sports exhibit higher psychological resilience than those in team sports, possibly due to the personal effort required to overcome difficulties inherent in individual competition. In addition, Yarayan et al. (2018) found that the psychological resilience of athletes varies according to the type of sport they engage in.

The observed difference in psychological resilience across age groups appears to increase with age. This increase in resilience is likely influenced by more excellent competition experience and accumulated expertise over time (Bülbül, 2015). Psychological resilience tends to increase with age due to the accumulation of life experiences, which provide individuals with a broader range of coping strategies to manage adversity. As individuals age, they often face and overcome various challenges, such as career pressures, personal relationships, and health issues, which build their capacity to adapt and recover from stress. Additionally, older individuals typically develop a greater sense of emotional regulation and a deeper understanding of their strengths and limitations, contributing to their ability to maintain psychological well-being under challenging situations. These cumulative experiences enhance their overall resilience, allowing them to navigate future challenges with more confidence and adaptability. Supporting this observation, several studies have found a positive correlation between age and psychological resilience (Kimhi et al., 2013; Ümlü and Recepoğlu, 2013; Kılıç, 2014; Karademir and Açak, 2019; Basım and Çetin, 2011; Kudaybergenova, 2022; Karabulut, 2024). However, other research does not report a significant relationship between age and resilience (Harrisson et al., 2002; Chan, 2003; Harvatin, 2009; Kahraman, 2016; Seçer, 2019; Darici, 2019; Karadeniz, 2023; Bülbül, 2015).

Regarding income, a significant difference was found between the challenge sub-dimensions of participants engaged in martial arts and the control, commitment, and challenge sub-dimensions of those who did not participate in sports. The discrepancy in findings regarding the relationship between income and psychological resilience could be attributed to the differing nature of the populations studied and the context in which the research was conducted. While some studies suggest that income may not directly impact resilience, others, like the current study, indicate that the benefits of sports participation may buffer the effects of moderate-income levels, leading to higher resilience. Engaging in martial arts, for instance, can foster qualities such as self-discipline and coping skills, which may enhance psychological resilience, irrespective of economic background, highlighting the potential of physical activities to cultivate resilience in diverse groups. Existing literature indicates a notable relationship between income level and psychological resilience (Gizir, 2007; Bahadır, 2009; Güngörmüş et al., 2015; Açikgöz, 2016; Durmuş, 2016; Aydöner, 2018). Specifically, Yöndem and Bahtiyar (2024) found that income level significantly influenced adolescent resilience. Similarly, Campbell-Sills et al. (2009), Helfrich et al. (2008), Hoşoğlu et al. (2018), Güngörmüş et al. (2015), and Parmaksız (2020), all identified a connection between income status and resilience in diverse groups. The current study finds that while income levels were moderate, participants who engaged in martial arts displayed higher levels of psychological resilience. This may be attributed to the positive psychological effects of sports participation, which can enhance resilience regardless of income status (Dishman et al., 2006; Babiss and Gangwisch, 2009; Duygu, 2022). Notably, some studies present contrary findings, where no significant relationship between income and resilience is observed (Gökçen, 2015; Topçu, 2017; Güler and Süslü, 2022; Kudaybergenova, 2022; Karadeniz, 2023).

An analysis of the participant’s scores on the psychological resilience scale, categorized by education level, revealed no significant differences between the control, commitment, and challenge sub-dimensions for female participants involved in martial arts. This finding is consistent with several studies in the literature, which report no significant relationship between education level and psychological resilience (Sivri et al., 2023; Karal and Biçer, 2020; Acar, 2008; Deniz et al., 2020; Gizir, 2007). However, a significant difference was observed between the education level and the control, commitment, and challenge sub-dimensions for participants who did not engage in martial arts. It may be explained by education’s role in shaping individuals’ cognitive and emotional resilience. Higher levels of education are often associated with improved problem-solving skills, greater access to resources, and enhanced social support networks, all of which can contribute to greater psychological resilience. Previous studies by Kudaybergenova (2022), Altıntop (2023), Sheard (2013), Yıldızdal (2002), and Ernas (2017) found a significant relationship between education level and psychological resilience.

Further analysis of the psychological resilience scale scores based on smoking status revealed that a significant difference was observed in the control sub-dimension among female participants who practiced martial arts. In the literature, Atan and Ünver (2019) reported a significant relationship between smoking and psychological resilience, with smoking being detrimental to resilience. Similarly, Kudaybergenova (2022) found a significant correlation between harmful substance use (including alcohol, cigarettes, etc.) and psychological resilience. These findings are corroborated by Gökçen (2015), who examined the psychological resilience levels of university students and found significant differences based on substance use status. Thus, the existing literature supports the results of the current study, reinforcing the negative impact of smoking and substance use on psychological resilience. No significant difference was observed between the smoking status variable and the commitment, control, and challenge sub-dimensions among participants who did not engage in martial arts. In a study examining the psychological resilience levels of nursing students, Yiğitbaş et al. (2018) reported no significant difference in smoking status.

An examination of the participants’ scores on the psychological resilience scale, categorized by the type of violence experienced, revealed no significant differences in the control, commitment, and challenge sub-dimensions for female participants involved in martial arts. However, an important difference was observed between the violence type variable and the control, commitment, and challenge sub-dimensions for participants who did not engage in martial arts. This relationship between the type of violence experienced and the psychological resilience scores was particularly evident in non-martial arts participants. The observed differences across these sub-dimensions suggest that exposure to various forms of violence (physical, emotional, sexual, etc.) could have a significant impact on how individuals experience and cope with psychological resilience, especially in non-physical domains.

For example, participants who have experienced different forms of violence may exhibit varying levels of resilience across control, commitment, and challenge. These variations reflect how trauma influences psychological processes, including emotional regulation, goal setting, and coping strategies. For instance, those who have experienced physical violence might show lower scores in the commitment sub-dimension, as they may be more prone to avoidance behaviors or have reduced motivation due to the emotional and psychological toll of trauma. Conversely, individuals exposed to emotional or sexual violence might demonstrate lower scores in the challenge sub-dimension, indicating difficulty in viewing adversity as an opportunity for growth due to past experiences of vulnerability.

In contrast, participants who engaged in martial arts training may benefit from the resilience-building elements inherent in such activities, which provide a more structured and supportive environment for coping with trauma. The physical and mental demands of martial arts can serve as protective factors against the negative psychological impacts of trauma, potentially mitigating the influence of violence exposure on resilience. However, for non-practitioners, the absence of such structured support could result in less effective coping mechanisms, explaining the observed differences in psychological resilience based on violence type.

This finding underscores the importance of considering the broader context of trauma when assessing resilience, especially for individuals who may not have access to structured support systems, such as martial arts training. Future research could explore the differential impact of specific violence types on resilience and examine how participation in different forms of physical activity may buffer or exacerbate the psychological effects of trauma. For instance, Gökmen (2009) found a significant relationship between women’s psychological resilience and the type of violence they experienced (e.g., physical, verbal, emotional). Furthermore, the physical damage caused by violence was identified as one of the most significant factors affecting the psychological well-being of women exposed to such experiences. Studies have shown that women who endure violence are at a higher risk of developing psychological disorders (Özyurt and Deveci, 2011), and they often seek medical attention for somatic, depressive, or anxiety-related symptoms (Akyüz et al., 2002).

### 4.1 Limitations and future research

This study has several limitations that should be considered when interpreting the findings. First, the cross-sectional design limits the ability to draw causal inferences about the relationship between martial arts participation and psychological resilience. While differences were observed across various demographic variables, the study cannot determine the directionality of these effects. Second, reliance on self-report measures introduces the potential for biases such as social desirability and recall inaccuracies, especially given the sensitivity of variables like exposure to violence and smoking behavior. Additionally, the sample selection poses several concerns. Although the study included women aged 18 and older who had been practicing martial arts (boxing, kickboxing, taekwondo, and Muaythai) for at least 3 years, the lack of psychometric testing for the sample limits the generalizability of the results. The low internal reliability of the commitment sub-dimension of the resilience scale further restricts confidence in interpreting subscale-specific differences. Moreover, including Pilates practitioners in the control group represents a potential confounding factor, as it introduces another form of physical activity that may influence psychological resilience independently of martial arts.

Future research should address these limitations by utilizing longitudinal or experimental designs to better capture changes in resilience over time. Incorporating physiological (e.g., cortisol levels, heart rate variability) or behavioral indicators of resilience would strengthen the measurement approach and reduce sole reliance on self-report data. Qualitative methods, such as in-depth interviews, could also offer richer insights into women’s lived experiences with martial arts and their coping strategies in the face of adversity and violence. Additionally, future studies could explore potential mediating or moderating factors such as social support, trauma history, or self-efficacy that may shape the relationship between martial arts engagement and psychological resilience. Including a more diverse and representative sample, both geographically and culturally, would also enhance the ecological validity of future findings.

### 4.2 Implications

The findings of this study suggest several implications for practice and policy. Women exposed to violence may benefit from participating in structured martial arts programs, which not only offer physical empowerment but also enhance psychological resilience. These programs can be integrated with social support services to create a more comprehensive intervention approach. Additionally, efforts to reduce smoking and alcohol use among women should incorporate resilience-building strategies, as healthier coping mechanisms may help mitigate psychological vulnerabilities. Socioeconomic support through inclusive social policies, such as improving access to education, employment, and financial assistance, can also play a critical role in strengthening women’s resilience. Psychological resilience training tailored to different educational levels may further help women develop more adaptive responses to stress and trauma. State-supported programs that provide safe, accessible, and culturally appropriate resilience-building courses for women exposed to violence are also necessary. Furthermore, public awareness initiatives that promote the psychological benefits of physical activity, particularly martial arts, could reduce stigma and encourage broader participation. Lastly, integrating resilience education into school and university settings may serve as a preventative measure, equipping young women with the tools to manage future adversity more effectively.

## 5 Conclusion

This study demonstrated that among female participants engaged in martial arts, psychological resilience was influenced by factors such as age, exposure to different types of violence, and smoking status. In contrast, psychological resilience was significantly associated with education level, age, and income for women not involved in martial arts. These findings contribute to the growing body of literature emphasizing the role of martial arts as a potential facilitator of psychological resilience in women. The results underscore the complex interplay between demographic factors and resilience, indicating that while some variables significantly influence, others may not yield measurable effects. Importantly, this study highlights the potential of martial arts to support the development of resilience, particularly for women who have experienced adverse life conditions. These insights inform future interventions and social programs that promote psychological well-being through sport-based engagement.

## Data Availability

The data that support the findings of this study are available from the corresponding author, upon reasonable request.

## Footnotes

^1^ https://docs.google.com
