# Correction: Building resilience through self-defense: the role of martial arts in enhancing psychological strength among women

**DOI:** 10.3389/fpsyg.2025.1655066

**Published:** 2025-08-06

**Authors:** Frontiers Production Office

**Affiliations:** Frontiers Media SA, Lausanne, Switzerland

**Keywords:** psychological resilience, women, violence, sport, martial arts

The affiliation for reviewer Jeong Hu Kim was incorrectly published as “University of Hertfordshire, United Kingdom.” The corrected affiliation is shown below:

“Catholic Kwandong University, Republic of Korea”

The original version of this article has been updated.

